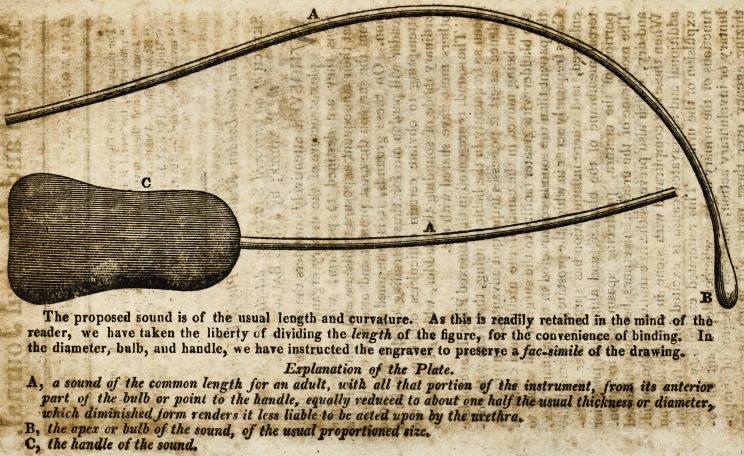# Sketch of an Improved Sound for Detecting the Stone in the Bladder

**Published:** 1816-02

**Authors:** James Barlow


THE LONDON

### Medical and Physical Journal.

2 OF VOL. XXXV.] FEBRUARY, 1816. [NO. 204.

"For many fortunate discoveries in medicine, and for the detection of nume-

"rous errors, the world is indebted to the rapid circulation of Monthly

"Journals; and there never existed any work to which the Faculty in

"EUROPE and AMERICA were under deeper obligations than to the

"Medical and Physical Journal of London, now forming a long, but an

"invaluable, series."—RUSH.

*For the London Medical and Physical Journal.*

### SKETCH *of an improved* SOUND *for detecting the* STONE *in the* BLADDER; by JAMES BARLOW, Esq.

WHOEVER attentively considers the delicate and complex structure of the urinary organs, and the functions they are destined to perform, will readily admit that their consequent exposure to disease and pain demands from the surgeon the most prompt and attentive consideration. On these grounds, it is presumed the experienced reader will pardon me for thus intruding on the public in attempting to obviate certain difficulties occurring in the ordinary mode of sounding the bladder, which, I presume, others must have shared with me.

The sound, represented in the annexed Plate, I have of late been so frequently in the habit of using, that I can with confidence assert it possesses important advantages in practice, from being moved in the canal of the urethra and cavity of the bladder with greater facility to the surgeon and safety to the patient than the sounds constructed in the ordinary way. Cases frequently occur in which the prostate gland is morbidly enlarged, and where inflammation has spread over the internal membrane of the bladder, and along the posterior portion of the urethra, producing spasm and irritation. The membrane of the urethra sometimes becomes inflamed and dry, which also prevents the free motion of the sound. When these are complicated with stone in the bladder, an additional and aggravated cause is excited for the frequent expulsion of the urine. This reiterated excitability of the functions of the urinary organs, whether dependant on voluntary or involuntary action, manifests a degree of sympathizing agency over these parts, and much increases the difficulty of introducing either the sound or catheter.

To accomplish the introduction of the sound, and search the cavity of the bladder with becoming dexterity, the surgeon geon should be furnished with a variety of instruments of different degrees of curvature, length, and diameter, proportioned to the age and bulk of the patient.

As no two cases are in every respect exactly similar, and as almost every writer on surgery has described the anatomy of the parts subservient to lithotomy, it may be presumed that the reader is fully acquainted therewith.

It will be granted that the management of the catheter requires nearly the same manœuvre to conduct it into the bladder as the sound. Let me then intreat the attention of the young surgeon to the concluding paragraph, so well expressed by an author of distinguished celebrity, when closing his directions on the mode of introducing this instrument into the bladder. "The catheter (says he) in the hands of a surgeon, like the pencil in the hand of a painter, requires frequent use, and much practice, to be managed with facility and success. Rules may be laid down for the forming a rough outline; but those more delicate movements, which, in many instances, are necessary to insure success, can no more be described, than a painter can describe those finer touches of his pencil which are necessary in the perfecting of some finished performance."

When a common-formed sound has been introduced into the bladder, for the purpose of exploring the different parts of that viscus to find the stone, I have frequently perceived the instrument so firmly embraced by the urethra, that it was scarcely moveable without the whole of that canal and body of the penis partaking of the motion exercised by the hand in attempting to complete this stage of the operation. Hence follow the difficulty and obscurity of such blind research, and the uncertainty of identifying the tremors communicated from the stone along the sound by the stroke of the instrument. These perverse circumstances first suggested to me the necessity of adopting a sound differently constructed from those in general use.

I am also disposed to believe that the failure of detecting the stone may not unfrequently be attributed to an irritable and spasmodic affection (independant on organic disease) of the muscles* of the perinæum and bladder opposing the movement of the instrument:—that, when its point is conducted to the membranous part of the urethra,

* Is it not an undue degree of action of the two muscles surrounding this portion of the urethra, as described by Mr. Wilson, which chiefly tends to impede the introduction of any instrument into the bladder? See Medico-Chirurgical Transactions, vol. i.

ejaculator seminis, and prostate gland, it excites the contiguous muscles of the lower part of the pelvis and bladder into action, and thus impedes the complete introduction of the instrument. In this uncertain and embarrassed posture of affairs, it is manifest that any movement of the sound, however judicious, must be fruitlessly employed, unless the stone be of considerable size, or seated near the neck of the bladder.*

The pain and spasm so frequently excited on the living body by the introduction of any instrument into the bladder when tortured with calculi, must have been noticed by most practitioners who are versed in this branch of surgery; and it is a fact which should never be overlooked by the junior surgeon, that a similar attempt to pass either the sound or catheter on the dead subject, is an operation of comparatively minor difficulty, when the muscles subservient to these parts have lost their vital energy.

Before dismissing this subject, I wish to mention that I have also been in the habit of occasionally sounding the urethra, to ascertain the place and extent of strictures, with this instrument, and sometimes using one made nearly strait; either of which excites less irritation, and is also passed along the canal of the urethra with greater facility than the bougie.

Having thus slightly marked out a few leading impediments in the mode of sounding the bladder, (which are scarcely noticed by surgical writers, when treating on the stone,) without entering into a laboured detail of every step and stage relative to the subject, I am induced to infer that the practical lithotomist will give me credit for having suggested an instrument which, when used with skill and adroitness, will surmount most of the incidental obstacles connected with the operation in question.

*Blackburn, Lancashire;*

*Dec.* 24, 1815.

---

*For the London Medical and Physical Journal.*

*On Midwives and Accoucheurs;* by JOSEPH ADAMS, M.D.

I HAVE been much interested in the controversy of some of your learned correspondents concerning the obstetric art, and shall feel obliged by your admitting a few loose

* In the course of an extensive practice, I have many times been able to detect the stone in the bladder, when other surgeons have repeatedly and fruitlessly employed all their efforts.

 thoughts

## Figures and Tables

**Figure f1:**